# Structure-Based Computational Study of Two Disease Resistance Gene Homologues (*Hm1* and *Hm2*) in Maize (*Zea mays* L.) with Implications in Plant-Pathogen Interactions

**DOI:** 10.1371/journal.pone.0097852

**Published:** 2014-05-21

**Authors:** Budheswar Dehury, Mahesh Chandra Patra, Jitendra Maharana, Jagajjit Sahu, Priyabrata Sen, Mahendra Kumar Modi, Manabendra Dutta Choudhury, Madhumita Barooah

**Affiliations:** 1 Department of Agricultural Biotechnology, Assam Agricultural University, Jorhat, Assam, India; 2 Department of Life Science and Bioinformatics, Assam University, Silchar, Assam, India; 3 BIF-Centre, Department of Bioinformatics, Orissa University of Agriculture and Technology, Bhubaneswar, Odisha, India; 4 Animal Genomics Laboratory, Animal Biotechnology Centre, National Dairy Research Institute, Karnal, Haryana, India; 5 Biotechnology Laboratory, Central Inland Fisheries Research Institute, Barrackpore, Kolkata, West Bengal, India; Russian Academy of Sciences, Institute for Biological Instrumentation, Russian Federation

## Abstract

The NADPH-dependent HC-toxin reductases (HCTR1 and 2) encoded by enzymatic class of disease resistance homologous genes (*Hm1 and Hm2*) protect maize by detoxifying a cyclic tetrapeptide, HC-toxin, secreted by the fungus *Cochliobolus carbonum* race 1(CCR1). Unlike the other classes' resistance (*R*) genes, HCTR-mediated disease resistance is an inimitable mechanism where the avirulence (*Avr*) component from CCR1 is not involved in toxin degradation. In this study, we attempted to decipher cofactor (NADPH) recognition and mode of HC-toxin binding to HCTRs through molecular docking, molecular dynamics (MD) simulations and binding free energy calculation methods. The rationality and the stability of docked complexes were validated by 30-ns MD simulation. The binding free energy decomposition of enzyme-cofactor complex was calculated to find the driving force behind cofactor recognition. The overall binding free energies of HCTR1-NADPH and HCTR2-NADPH were found to be −616.989 and −16.9749 kJ mol^−1^ respectively. The binding free energy decomposition revealed that the binding of NADPH to the HCTR1 is mainly governed by van der Waals and nonpolar interactions, whereas electrostatic terms play dominant role in stabilizing the binding mode between HCTR2 and NADPH. Further, docking analysis of HC-toxin with HCTR-NADPH complexes showed a distinct mode of binding and the complexes were stabilized by a strong network of hydrogen bond and hydrophobic interactions. This study is the first *in silico* attempt to unravel the biophysical and biochemical basis of cofactor recognition in enzymatic class of *R* genes in cereal crop maize.

## Introduction

Plant diseases can considerably decline not only the net crop yields but also the crop quality by releasing toxins that affect human health, as the outcome of disease outbreak is getting severe across the globe. The nature has blessed the crop plants with an inherent mechanism to defend themselves from the invasion of pathogens, termed resistance, which restricts further incursion and proliferation of potential pathogens. The complex network of inherent defense system in plants is comprised of three steps that include pathogen detection, signal transduction, and defense response initiation [1]–[3]. Induction of defence response involves recognition of specific pathogen effectors by specialized host genes, called resistance (*R*) genes. The host plant then initiates transcription of the defense response (DR) gene, including the pathogenesis-related (*PR*) gene that confers local or systemic resistance [4], [5].

Because of selective pressure from multitude of pathogens, plants have evolved post invasion mechanisms, which are controlled by dominant resistance genes that detects specific pathogen effector molecules (for example, Avirulence molecule (Avr)) through direct or indirect means and initiates active defense response. The R-gene mediated resistance is fundamentally race-specific which is only effective against pathogen strains expressing the cognate effector recognised by the R protein. This mechanism is frequently associated with hypersensitive response (HR), resulting in death of the infected cells, also known as gene-for-gene (R-Avr) interaction.

Apart from the major classes of R genes (NBS, LRR, TLR, CC, Kinase etc.), the enzymatic R-genes provide exceptional resistance to the plants. The two structurally homologous disease resistance genes, *Hm1* and *Hm2*, represent two unique subtypes of the enzymatic R-gene class in the cereal crop maize [6]–[8].

In maize, the leaf blight disease caused by the fungus *Cochliobolus carbonum* race 1 (CCR1) affects net yield potential. The asexual form (i.e., *Helminthosporium carbonum* (HC)) is the most destructive biotic fungal pathogen that kills susceptible maize plants at any stage of development [9]. Unlike other plant pathogens, CCR1 affects every part of the host causing blight of the leaves, rot of the roots and the stalk, and mold of the ear. In maize the R gene *Hm1* provides complete protection against southern leaf blight caused by CCR1. *Hm1* encodes a nicotinamide adenine dinucleotide phosphate (reduced form of NADPH)-dependent enzyme HC toxin reductase (HCTR), which detoxifies the key virulence factor HC toxin − a specific cyclic tetrapeptide toxin produced by the CCR1 [10]. In contrast to other classes of *R* genes, *Hm1 encoded* HCTR does not interact with the *Avr* component of CCR1 in a gene-for-gene manner, and this could be thought as a natural selection in maize. *Hm1* was the first DR gene to be cloned, which disarms the pathogen directly instead of participating in the plant recognition and response system as most DR genes do. Furthermore, *Hm1* is found to be conserved in all monocots including rice, barley, and sorghum [11]. Interestingly, orthologs of *Hm1* are present in the grass family, though CCR1 is an obligatory pathogen of maize, suggesting an ancient evolutionarily origin this DR trait in plants.

Apart from *Hm1* gene, certain lines of maize contain a second DR gene named *Hm2*, which confers effective resistance only in adult plants. Both *Hm1* and *Hm2* encode nitrate reductases that detoxify the HC-toxin of CCR1 [12]. In addition, *Hm2* encodes a structurally truncated duplicate of *Hm1* [13]. However, the functional efficiency of *Hm2* is quite different from *Hm1*. Both these genes are different in two aspects; *Hm1* is completely dominant conferring absolute resistance to plants, whereas *Hm2* exhibits incomplete dominance. The former provides absolute protection in all parts of the plant at all stages of development, while the later confers effective resistance only at maturity. Thus, the dominant nature of *Hm1* masks the role of *Hm2* in the maize germplasm. Nevertheless, *Hm2* retains its efficacy in *Hm1* knock-out plants.

The NADPH-dependent HCTR enzymes show striking homology with many secondary metabolite biosynthesis enzymes of plants including dihydroflavonol reductase (DFR), vestitone reductase, and anthocyanidin reductase. NADPH plays a major role in cellular redox homeostasis in plants, and is an indispensable electron donor in numerous enzymatic reactions, biosynthetic pathways, and detoxification processes [9]. Although several proteins encoded by the diverse set of resistance genes have been characterised till date, the structural and functional analysis of *Hm1* and *Hm2* remain elusive. Recently, for the first time, we have reported our preliminary findings on the mode of cofactor binding in the *Hm1* encoded HCTR1 of maize [14].

In the present study, we have used comparative modeling and molecular docking methods to propose a structural model for ligand recognition by NADPH-dependent HCTRs. In order to better understand the mechanism of cofactor binding, the modeled HCTRs were docked with NADPH and analyzed by molecular dynamics (MD) simulations and molecular mechanics/Poisson-Boltzmann surface area (MM/PBSA) binding free energy calculations. Further, the HC-toxin was docked near the cofactor binding site and critical residues responsible for ligand binding were identified. We expect translation of these findings into other economically important crop species will have a significant contribution in exploring similar genes for achieving more durable resistance against pathogens. This is the first *in silico* structural-biology prospective to unravel the critical residues those aid in cofactor and HC-Toxin recognition by enzymatic class of disease resistance genes in an important cereal crop like maize.

## Materials and Methods

### Sequence retrieval and bioinformatics analysis

The reviewed full length cDNAs of *Hm1* [9], [10], [15] and *Hm2* [12] genes of maize were downloaded from GenBank database of NCBI. The cDNAs of *Hm1* and *Hm2* (GenBank accession numbers: NM_001112450 and EU367521) represent 357 and 360 amino acids of HCTR1 and 2, respectively. The putative conserved domains and families of HCTRs were identified using Pfam [16] database implemented in SMART [17]. In addition, InterProScan [18] was used for predicting the protein family, superfamily, and the domain arrangement within both the HCTRs.

### Comparative modeling of HCTRs

The search of suitable templates for both the maize HCTRs was performed using DELTA-BLAST [19] against Protein Data Bank (PDB). The search considered the following parameters: substitution matrix, BLOSSUM62; gap opening penalty, −500; gap extension penalty, −50; and e-value threshold, 5. As the resulting templates shared poor sequence identities (that is below the cut-off of ∼30%) with our target sequences, the template search was carried out using various protein fold recognition servers that included Gensilico metaserver2 [20], Phyre (Protein Homology/analogY Recognition Engine) V 2.0 [21], I-TASSER [22], and SPARKS-X [23]. The fold recognition servers suggested the same templates as identified through DELTA-BLAST search for both the HCTRs. Thus, with a consensus, we chose the templates with PDB IDs: 2C29-D [24], 2RH8-A [25], and 2P4H-X [26] for constructing 3D models of HCTRs using MODELLER 9.12 [27] software. A total of 200 models for each HCTR sequence were generated and were ranked according to their discrete optimized potential energy (DOPE) scores. The model with lowest DOPE score and least restraints violations was selected for further modeling exercises. To ensure the correctness of the MODELLER-derived models, automated modeling servers viz., (PS)^2^ [28], LOMETS [29], Phyre2 [21], and I-TASSER [22] were also used for comparison. The best HCTR models were subjected to loop refinement using Looper algorithm implemented in Discovery Studio 3.5 (DS3.5; Acclerys software *Inc*., CA, San Diego, USA). Finally, the models were energy minimized using GROMACS 4.6.4 [30] simulation package to relieve atomic close contacts.

### Model evaluation and quality assessment

After initial round of energy minimization, the refined models of HCTRs were subjected to structural evaluation and stereochemical quality assessment using PROCHECK [31], ERRAT [32], Verfiy 3D [33] and PROVE [34] programs integrated in Structural Analysis and Verification Server (SAVeS) (http://nihserver.mbi.ucla.edu/SAVES/). The native folding of the modeled HCTRs were assessed using Protein Structure Analysis (ProSA) [35] tool. The bond length and bond angle analysis of the modeled structures were performed using MolProbity [36]. The Z-score of hydrogen bond (H-bond) energy, packing defect, radius of gyration (Rg) and deviation of Ω angles of the refined models were tested in VADAR [37]. The overall stereochemical qualities of the models were predicted through ProQ [38] and ModFOLD v4.0 [39].

### Identification of Cofactor binding site on HCTRs

The active site pockets of the modeled HCTRs were predicted using CASTp server [40]. In addition, GalaxySite tool of GalaxyWEB server [41] was employed to predict the ligand and cofactor binding sites. Further, COFACTOR tool (an award-winning method for function prediction in the community-wide CASP9 analysis, 2010) was used for functional annotation of the modeled HCTRs [42]. To ensure the accuracy of the predicted binding pockets, the closest structural homolog of both HCTRs *i.e.*, DFR of grape (PDB ID: 2C29, D chain) was superposed. The NADPH binding residues of HCTRs thus identified were compared with the residues predicted by the CASTp, GalaxySite, and COFACTOR. This way, the consensus binding site residues for both the HCTRs were ascertained.

### Molecular docking of HCTRs with the cofactor NADPH

The cofactor NADPH was docked into the active site of the modeled HCTRs to elucidate the intermolecular interaction and recognition specificities. The CDocker [43] module of DS3.5 was employed to construct the receptor-cofactor complexes and to assess the binding specificity of the NADPH within the active site of modeled HCTRs. The binding site was defined with a 12 Å grid radius that was large enough to cover the binding pocket. HCTRs were kept rigid while NADPH was flexible during the docking calculation. The initial ligand structure, obtained from the template (DFR of grape), was prepared using ligand preparation protocol of DS3.5. A number of NADPH conformations were generated through high temperature molecular dynamics, followed by random rotations. The random conformations were refined by simulated annealing and a final energy minimization.

The refined HCTRs were prepared by removing water molecules and subsequently adding hydrogen atoms. The binding affinity of NADPH was measured using CDOCKER energy, interactions of ligand poses (H-bond count and contact count) and root mean square deviation (RMSD) calculation in protein-ligand interaction module of DS3.5. Finally, of the resulting 30 docking poses for both the HCTR models, the one with desired orientation of the carbonyl group close to NADPH was used for further energy refinement and binding energy calculation. The best complexes were subjected to MD simulations to optimize the enzyme-cofactor interactions.

### MD simulations of HCTR-NADPH complexes

MD simulations were performed to assess the structural integrity of the docked complex between HCTRs and NADPH. All simulations were performed using TIP3P water model and GROMOS96 43a1 force field [44] for protein in GROMACS 4.6.4 package. Each model was surrounded by a periodic box that extends 11 Å from the protein atoms. The protonation states of all the ionizable amino acids were determined at pH 7.0. To neutralize the system, sodium counterions were added replacing random water molecules. The atomic composition of the simulation systems is listed in Table S1 in File S1. The Energy minimization was performed using steepest descent algorithm for 10,000 steps. A 1-ns position restrained and a 30-ns production MD simulation was performed for each simulation system at constant pressure (1 bar) and temperature (300 K). Covalent bonds in the enzymes and water molecules were constrained using the SHAKE and SETTLE algorithms, respectively. A twin cut-off scheme of 9 Å was implemented for treating long-range and van der Waals interactions. Electrostatic interactions were computed using the particle mesh Ewald (PME) method. The time step for MD simulation was 2 fs and the snapshots were saved every 1 ps. The trajectory analysis was performed using visual molecular dynamics (VMD 1.9.1) and Grace 5 (http://plasma-gate.weiz-mann.ac.il/Grace/) programs. All computations were conducted with a high performance computer cluster.

### Binding free Energy Calculation

A total of 500 snapshot structures were extracted from the 30-ns dynamics trajectories of each HCTR-NADPH simulation system at a time interval of 60 ps. The binding free energies (ΔG_binding_) were estimated using GMXAPBS tool [45], which implements MM/PBSA method [46]–[47] as shown in eqn.1. 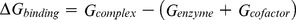
(1)


The free energy calculations of the individual components were performed as follows:
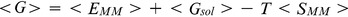
(2)


The molecular mechanics interaction energy, E_MM_ is defined as:
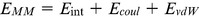
(3)


Where E_int_ denotes bond, angle, and torsion angle energies, E_coul_ indicate electrostatic energy, and E_vdW_ represents van der Waals energy.

The solvation free energy term, G_sol_ is divided into polar and nonpolar contributions:
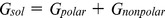
(4)

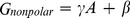
(5)


In this study, the G_polar_ and G_nonpolar_ terms were calculated using APBS program [48]. The polar term (G_polar_) was calculated by solving nonlinearized Poisson–Boltzmann (PB) equation. The parameters employed for APBS calculation were as follows: grid spacing, 0.5 Å; temperature, 296 K; and salt concentration, 0.15 M. The surface or nonpolar solvation term G_nonpolar_ is defined as the solvent accessible surface area, A, and two empirical parameters γ = 0.0227 kJ mol^−1^ Å^2^ and β = 0 kJ mol^−1^. Here, A was estimated using the Shrake-Rupley numerical approximation implemented in the APBS package. The dielectric boundary was set with a probe radius of 1.4 Å. The free energy calculations were carried out using the single trajectory method, which provides fairly good estimates for the relative binding energies [46]. The standard errors (SE) were computed using following equation:
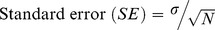
(6)


Where σ is the standard deviation and N is the number of structures used in the calculation.

### Docking of HC-toxin with the complex of HCTR-NADPH

To explore the critical residues of HCTRs involved in recognizing the HC-toxin we docked the chemical structure of HC-toxin into the already docked HCTR-NADPH complexes using Autodock 4.2 [49]. The 2D structure of HC-Toxin (3,6-dimethyl-9-[6-(oxiran-2-yl)-6-oxohexyl]decahydropyrrolo[1,2-a][1], [4], [7], [10]tetraazacyclododecine-1,4,7,10-tetrone: CID 3571) was obtained from NCBI's PubChem database (http://pubchem.ncbi.nlm.nih.gov/). The obtained 2D coordinates were converted into 3D coordinates using Automated Topology Builder (ATB) server [50] followed by energy minimization with Gromos96-53a6 force field. Lack of experimental evidence on catalytic sites of HCTRs possesses a constraint to elucidate the probable binding pocket of HC-Toxin. We made an assumption that HC-Toxin must bind the enzyme alongside the cofactor in physiological condition. The binding site grid was centered on the already docked NADPH with a grid dimension of 90×90×90 grid points and 0.375 Å of grid spacing that almost covered the whole protein.

## Results and Discussion

### Domain architecture analysis

The SMART search revealed that HCTR1 (357 amino acids) possesses four overlapping putative domains *viz*., NmrA (Val11-Cys119), Epimerase (Val11-Cys278), 3Beta_HSD (Cys12-Leu207) and NAD binding 4 domains (Val13-Leu262). HCTR2 (360 amino acids) consists of five domains, namely short chain dehydrogenase (Val5-Gly139), Epimerase (Val7- Ala275), NmrA (Val7-His125), 3Beta_HSD (Cys8 -Ser267) and NAD binding 4 domain (Val9 to His259). These cl09931 superfamily of proteins are comprised of Rossmann-fold NAD(P)(+) binding proteins sharing a Rossmann-fold NAD(P)H/NAD(P)(+) binding (NADB) domain, found in numerous dehydrogenases and redox enzymes with a vital role in several metabolic pathways and detoxification processes. In addition, these reductases contain a second domain involved in binding of substrates and catalysis of a particular enzymatic reaction. Although, HCTR2 is a truncated homolog of HCTR1, both these enzymes share a sequence similarity of 58.84% and an identity of 46.44%.

### Comparative modeling and validation of modeled HCTRs

**Figure 1:**
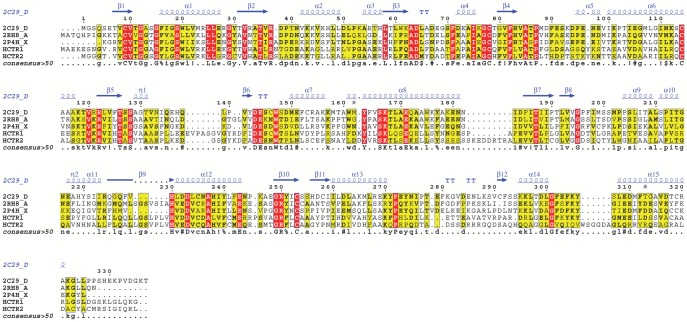
Multiple sequence alignment between the HCTRs (HCTR1 and 2) and the templates constructed using Clustal Omega and rendered using ESPript. The secondary structural elements were identified from the crystal structure of DFR of grape. The α-helices, 310 (η)-helices, β-sheets and strict β-turns are denoted α, η, β and TT respectively. The gray stars indicate side chains for which multiple conformations were modeled. Similar amino acids are highlighted in yellow square boxes, and completely conserved residues are indicated by white lettering on a red square boxes. ^*^PDB IDs: 2C29 is the crystal structure of DFR of grape; 2RH8: apo anthocyanidin reductase of grape (*Vitis vinifera*) and 2P4H: vestitone reductase from Alfalfa (*Medicago sativa* L.).

The 3D models of HCTR1 and 2 were constructed based on the crystal structures of DFR of grape (*Vitis vinifera* L., PDB ID: 2C29), Vestitone Reductase of Alfalfa (*Medicago sativa* L., PDB ID: 2P4H), and Apo Anthocyanidin Reductase of grape (PDB ID: 2RH8). Pairwise alignment (Figure 1) revealed that HCTR1 had a sequence identity of 29, 31, and 30% with 2C29, 2P4H, and 2RH8, respectively. Similarly, HCTR2 shared a sequence identity of 29, 28, and 29% with 2RH8, 2C29, and 2P4H, respectively. The stereochemical quality parameters and other validation scores of the models have been described in Table 1, and Figure S1 and Text S1 in File S1.

**Table 1:** Comparison of the stereochemical quality of homology modeled HCTR1, HCTR2 and closest structural homologue (crystal structure of DFR of grape: 2C29 chain D).

Model validation Servers	Model Validation Scores	HCTR1	HCTR2	2C29_D
Procheck	Most favored regions (%)	90.40	90.20	91.00
	Additionally allowed regions (%)	8.30	8.60	8.30
	Generously allowed regions (%)	1.30	1.30	0.70
	Disallowed regions (%)	0.00	0.00	0.00
	Overall G-factor	0.19	−0.14	0.14
Verfiy-3D	Averaged 3D-1D Score >0.2	95.81	94.97	99.69
ERRAT	Overall Quality (%)	71.38	78.90	97.42
PROVE	Prove RMS Z-score	0.75	0.09	0.28
ProSA	Z-score	−8.41	−7.12	−10.93
ProQ	LG score	5.88	7.23	6.23
	MaxSub	0.24	0.80	0.27
MolProbity	Residues with bad bond lengths	0.40	−0.33	−0.33
	Residues with bad angles	1.98	1.81	−0.69
	Clash score	0.59	0.79	0.48
Vadar (Z score)	Standard deviation of χ1 pooled	−8.60	−8.60	0.64
	Mean H-bond energy	8.35	8.75	0.31
	Generously allowed Ω angles (%)	−1.60	−1.60	−1.60
	Packing defects (%)	−2.18	−2.18	0.86
ModFold	Global model quality score	0.74	0.74	0.94
	p-value	4.197E-4	3.96E-4	5.067E-5

### Overall structure of modeled HCTRs

**Figure 2:**
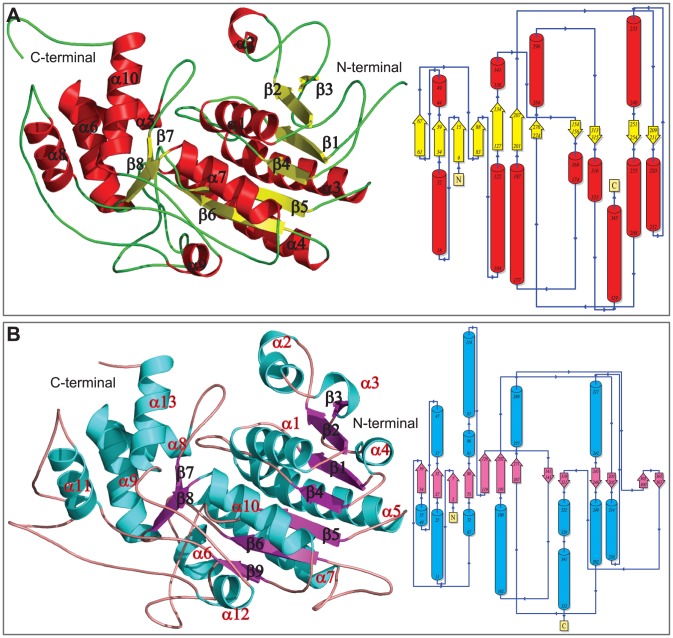
The overall 3D structures of modeled HCTR1 and 2 of maize. The secondary structure elements were assigned using Pymol.

The predicted model of HCTR1 consists of two domains: a long N-terminal domain (the dinucleotide binding domain) adopting a classic Rossmann fold [51], and a C-terminal substrate binding domain where the active site lies within the deep cleft formed by the two discrete domains. The Rossmann fold consists of seven β-strands forming a large parallel β sheet flanked by seven α helices and this domain is stabilized by four β-α-β units (key functional units in reductase enzymes) (Figure 2A). The retention of a higher number of β-α-β folds is one of the characteristics features of NADPH and NADH dependent reductases, which was also reported to be present in the crystal structure of DFR of grape [24]. However, unlike the DFR of grape, presence of a single β strand and one α helix within the Rossmann fold disrupts the overall symmetry of the two halves of β-α-β-α-β fold in HCTR1 (Figure 2A). In contrast, the small substrate binding domain is comprised of six α helices and four parallel β strands. The modeled HCTR2's architecture was somewhat different where 52 amino acids (14.6%) formed strands, 134 amino acids formed (37.5%) helices, and the rest 171 amino acids (47.9%) formed other secondary structure elements (turns/coils). Similar to HCTR1, the N-terminal domain of HCTR2 adopts a Rossmann fold with GXGXXG motif for NADPH binding with four β-α-β motifs. A profound variation was observed in the C-terminal domain of HCTR2, where the numbers of helices were more along-with one β hairpin joining an adjacent β-sheet as compared to HCTR1 (Figure 2B).

To comprehend the active site architecture of the modeled HCTR enzymes of maize, the pair-wise 3D structural superposition with DFR of grape (PDB ID: 2C29) was performed using MATRAS server. The best structural superposition with a RMSD of 0.8 Å on Cα atoms is shown in Figure S2A in File S1. Similarly HCTR2 also showed a very low RMSD of 0.5 Å with 2C29 as compared to the other two templates (Figure S2D in File S1). Furthermore, when the modeled HCTR1 and 2 were superimposed over each other using Cα atoms, the RMSD was found to 0.35 Å, which indicated that both HCTRs shares the common structural features as that of DFR of grape. As in the crystal structure of DFR, both HCTRs are comprised of the two active pockets: a cofactor binding pocket and a substrate binding pocket (a common characteristics seen in almost all the NADPH dependent reductases). Moreover, the NADPH binding region (GxGxxG motif) and the substrate binding channel were well conserved. Although both HCTRs superimpose very well with crystal structure of DFR, a minute variation occurs close to the shift of the chain around the substrate binding site, which is thought to be the sole factor toward diverse substrate specificities of these reductases.

### Identification of active site and molecular docking

Structure superimposition of the modeled HCTRs over 2C29 (the template) revealed the probable active site residues in the HCTRs. For HCTR1, the residues Gly18, Phe19, Ile20, Arg40, Lys47, Asp68, Leu69, Val88, Thr90, and Val210 were found to form the active site. However, some of the active site residues of DFR *i.e.*, Ser14, Tyr163, Lys167 and Ser205 showed variation with respect to the corresponding positions in the modeled HCTR1. The residues Ser14 and Ser205 of the template are replaced by Ala17 and Thr222 in the modeled enzyme. Similarly, for HCTR2 the active site residues were found to be Ser10, Gly11, Leu13, Arg33, Lys40, Asp60, Val80, Thr82, Tyr165, Lys169, and Val204). However, Tyr12, Met61, Asn216 were found to be variable with respect to Phe16, Leu65 and Ser205 of DFR. Among the catalytic residues, only Ser128 of DFR was found to be conserved whereas other four residues *viz.,* Phe152, Lys156, Tyr163, and Lys167 showed great variation, suggesting the catalytic mechanism of DFR and HCTRs may be different. Docking studies revealed that the cofactor was docked deep inside the cleft formed by the N-terminal large and small substrate binding domains. The active site residues of the HCTRs formed a strong network of H-bond and hydrophobic interactions with NADPH, as summarised in Table 2 and Figure S3 in File S1. Different energy components involved in cofactor recognition as derived from docking calculation is listed in Table S2 and S3 in File S1. The method for cofactor conformation generation and the details of scoring functions used in the docking calculation are described in Text S2 and Table S4 in File S1.

**Table 2:** Comparative analysis of interaction of cofactor (NADPH) with HCTR1 and HCTR2 before and after MD simulation.

Before Simulation						After Simulation					
(A) HCTR1-NADPH											
Hydrogen Bond			Hydrophobic Interaction	Electrostatic Interaction	Van der Waals Interaction	Hydrogen Bond			Hydrophobic Interaction	Electrostatic Interaction	Van der Waals Interaction
HCTR1	NADPH	length	Ala17	Gly15	Leu69	HCTR1	NADPH	length			
Arg40:HH22	O47	1.9	Ile20	Thr38	Phe92	Phe19:H	O2	2.0	Ala17	Gly18	Phe70
Val88:O	H6	2.4	Val88	His130	Ala136	Arg40:HE	N15	2.3	Ile20	Thr131	Leu87
Gly208:O	H5	2.5	Ala89	Thr131	Pro139	Thr90:H	O3	2.1	Ala132	Ser133	Val88
Arg218:HH12	O9	1.9	Ala132	Ser133	Leu209	His130:HE2	O40	1.6		Gly217	Ala89
Arg218:HH22	O11	1.7	Val210	Gln184		Thr131:O	H34	2.5		Pro220	Pro91
Arg218:HH21	O48	2.0		Gln187		Gln187:HE22	O40	1.7			Phe92
Glu221:NH	O25	2.3		Leu207		Arg218:HH21	O47	2.1			Val108
				Gly208		Arg218:HE	O46	2.3			Leu183
						Arg218:H	O46	1.8			Ala219
						Arg218:O	H9	2.3			
						Thr222:H	O25	1.9			
											
(B) HCTR2-NADPH											
HCTR2	NADPH	length				HCTR2	NADPH	length			
Tyr12:NH	O3	2.3	Tyr12	Gly8	Val80	Ser10:HG	O47	1.6	Tyr12	Gly9	Gly11
Leu13:NH	O25	2.1	Ala124	Gly11	Ala81	Leu13:H	O32	2.3	Ala81	Ser10	Leu32
Lys40:HZ1	O47	2.1		Gly14	Leu84	Arg33:H	O46	1.8	Val126	Gly14	Leu84
Ala59:O	H3	2.7		Thr31	Ala128	Ser34:H	O48	2.2	Tyr165	Pro83	Val80
Thr123:O	H7	2.4		Asp60	Cys201	Lys40:HZ1	O48	2.3		Thr123	Thr127
Lys169:NH	O40	2.4		Met61	Val204	Thr82:HG1	O2	2.0		Cys201	Gly202
Gln210:HE22	O3	2.2		Thr123	Trp214	Thr82:HN	N15	2.0		Gly215	Val204
Asn216:NH	O32	2.2		Ser125		Ala124:O	N41	2.3			Trp214
				Gly215		Ser125:HG	O40	1.5			
						Tyr165:HH	O40	2.5			
						Lys169:HZ1	O40	2.2			
						Gln210:HE22	O26	2.7			
						Asn216:HD22	O26	2.3			

#### Stability of and flexibility of Enzyme-cofactor complexes

**Figure 3:**
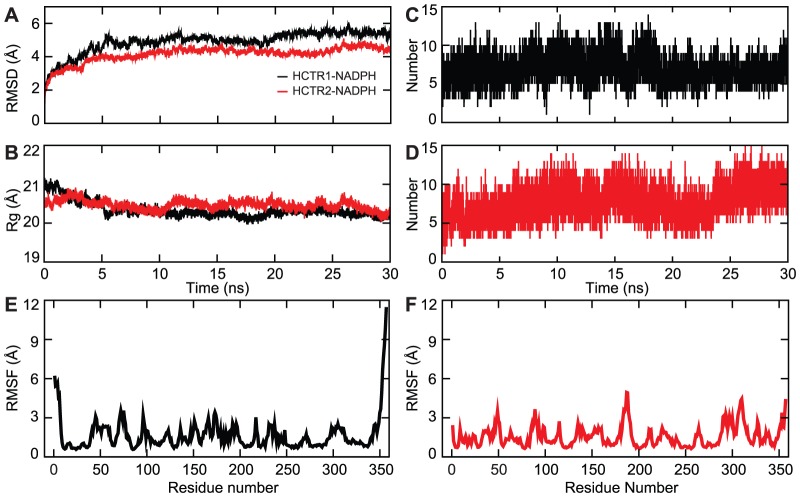
The stability parameters of the HCTR1-NADPH and HCTR2-NADPH complexes during 30-ns MD simulation. (A) RMSDs (B), Radius of gyration, (C) Total number of intermolecular H-bonds formed between HCTR1 and NADPH (D) Total number of intermolecular H-bonds formed between HCTR2 and NADPH (E) RMSF of HCTR1-NADPH complex (F) RMSF of HCTR2-NADPH complex. Black and red colors represent HCTR1-NADPH and HCTR2-NADPH complexes, respectively.

The 30-ns MD simulations were performed on HCTR1- NADPH and HCTR2–NADPH complexes. Both the enzyme-cofactor systems were found to be stable throughout the simulation, which was ascertained by observing their RMSD values as a function of simulation time. The average RMSDs of both the complexes were found to be ∼4.5 Å (Figure 3A), which remained largely constant soon after first 2 ns simulation, signifying that the modeled structures do not deviate unnaturally during MD simulation. Moreover, the potential and total energies of both the systems were stable after 1.2 ns. The persistent gyration radii of 20.5 and 20.2 Å for HCTR1-NADPH and HCTR2-NADPH respectively revealed that both the systems maintained a consistent shape and size during MD simulation (Figure 3B). The averaged root mean square fluctuations (RMSF) for HCTR1-NADPH and HCTR2-NADPH complexes were 1.67 and 1.78 Å, respectively. As can be seen from Figure 3E and 3F, the Cα RMSF of HCTR2-NADPH is larger than that of HCTR1-NADPH, which implies that HCTR2-NADPH complex undergoes greater conformational alterations after complex formation. The RMSF curves clearly signify that although HCTR2-NADPH has the largest fluctuations, the pattern of RMSF deviation in both the systems is overall the same. The secondary structure elements were found to be highly stable during the 30-ns of MD simulations as shown in Figure S4 in File S1.

#### Hydrogen bond analysis between HCTRs and NADPH

In order to understand the nature of cofactor binding in HCTR1 and 2, the numbers of intermolecular H-bonds formed during the simulations were calculated as a function of time. Both the complexes showed a constant H-bond interaction throughout the simulation, which gives direct clues about the cofactor's strong affinity towards the enzyme (Figure 3C and 3D). Although minute fluctuations in the number of H-bonds were observed, the interactions of key residues were conserved. The average number of H-bonds in HCTR1-NADPH complex was found to be 11 and that in HCTR2-NADPH was 9.

**Figure 4:**
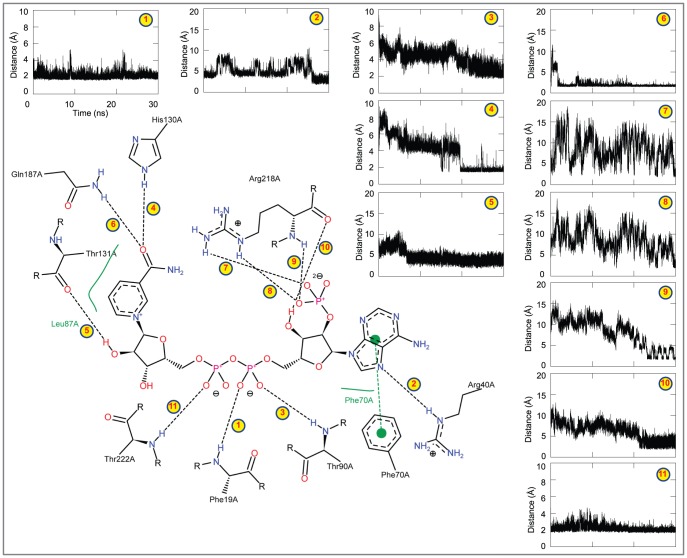
Snapshot of the H-bonds formed between NADPH and HCTR1. The figure shows the intermolecular H-bonds formed between HCTR1 and NADPH in the final representative structure obtained in the end of 30-ns MD simulation. The figure accompanies the distance of each observed H-bond.

The final representative structure of HCTR1-NADPH complex showed a maximum of 11 bonding-bonds (Figure 4). The distances between NADPH and the constantly H-bond forming residues were measured as a function of time (Figure 4). The constantly H-bond forming residues in HCTR1-NADPH complex include Phe19, Arg40, Thr90, Gln187, Arg218, and Thr222and the average interatomic distance between NADPH and these residues were below 2.5 Å, which signifies their importance in maintaining the overall stability of the enzyme-cofactor complex. Apart from main chain interactions, the side of chain HE atom of Arg40 interacts with nitrogen (N15) atom of NADPH (Table 2A). Similarly, HH21 and HE atoms of Arg218 interact with phosphate-oxygen atoms (O47 and O48) of the NADPH with interatomic distances of 2.1 and 2.3 Å, respectively. Furthermore, HE2 and HE22 atoms of His130 and Gln187 form two H-bonds with oxygen (O40) atom of NADPH. Altogether, the average interatomic distance was below 2 Å with a minimal standard deviation indicating the importance of H-bond forming residues in holding the NADPH in proper orientation at the active site of HCTR1(Figure 4).

**Figure 5:**
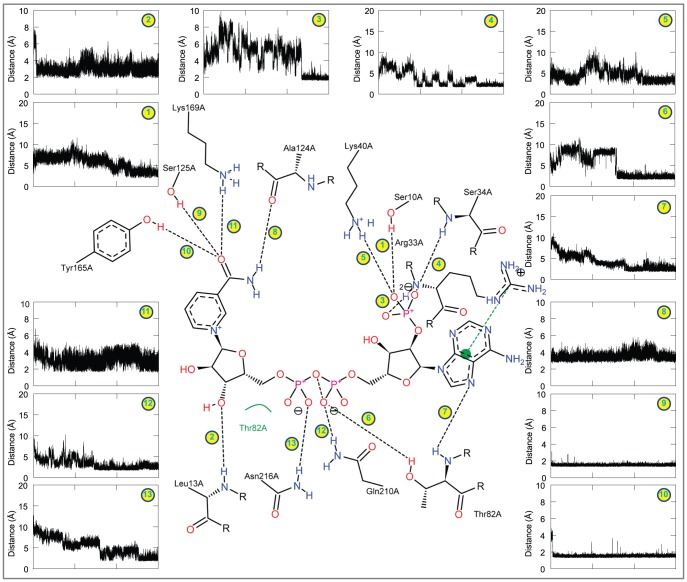
The H-bonds observed between NADPH and HCTR2. NADPH forms 13 H-bonds with the active sites of the HCTR2. The H-bond distances were plotted as a function of time and are indicated by a number corresponding to the observed H-bond in the figure at the center.

Analysis of the final representative structure of HCTR2-NADPH complex revealed a total of 13 intermolecular H-bonds between NADNPH and the key residues Ser10, Leu13, Arg33, Ser34, Lys40, Thr82, Ala124, Ser125, Tyr165, Lys169, and Asn216 (Table 2B and Figure 5). Importantly, most of the H-bonds occur via side chain contacts with an average interatomic distance of ∼2.13 Å (Figure 5 and Table 2B). As in HCTR1, the average interatomic distance was below 2 Å with a minimal standard deviation reflecting the importance the H-bond forming amino acids in holding the cofactor in its suitable orientation and position within the active site of HCTR2 (Figure 5). Despite the fact that the H-bonds in both the complexes equilibrate between formed and broken states during the course of the simulation, the cofactor remained tightly bound in the active site pocket. This suggests that other potentially relevant interactions (*i.e.*, electrostatic and van der Waals) compensate the loss of H-bonds, thus stabilizing the ligand. The detailed comparison of intermolecular association between enzyme and the cofactor before and after MD simulation has been summarized in Table 2. The continuously H-bond forming residues of HCTR1 and 2 are listed in Table S5 in File S1.

#### MM-PBSA free energy analysis for the wild-type complexes

**Figure 6:**
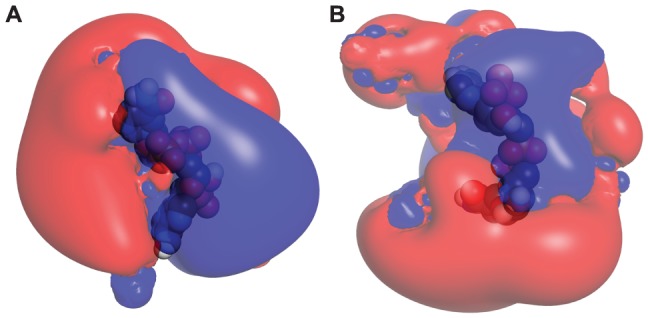
Electrostatic surfaces surrounding NADPH in HCTR1 and 2. Electrostatic surface potentials of (A) HCTR1-NADPH, and (B) HCTR2-NADPH complexes (only residues around 4 Å of NADPH have been modeled). The red and blue coulombic cages represent negatively and positively charged surfaces, respectively. The NADPH has been shown in vdW representation.

To characterize the strength of interaction between HCTRs and NADPH, MM/PBSA binding free energy calculations were performed on a total of 500 snapshots extracted from the 30-ns MD trajectories (see materials and methods). The decomposition of binding free energy terms are listed in Table 3. The overall binding free energies of HCTR1-NADPH and HCTR2-NADPH were calculated to be −616.989 and −16.9749 kJ mol^−1^, respectively. This indicates that the HCTR1-NADPH complex is energetically more stable than HCTR2-NADPH. The nonpolar contribution seemingly plays a decisive role for cofactor binding in HCTR1 and is influenced mostly by the van der Waals interaction energy. The presence of large number of hydrophobic residues of HCTR1 around NADPH could result in the increased nonpolar contribution to NADPH binding. The electrostatic as well as the polar solvation energy does not contribute to NADPH binding in HCTR1. This suggests that the loss of electrostatic interaction in HCTR1-NADPH might have been compensated by the van der Waals. On the other hand, electrostatic terms play dominant role in stabilizing the binding mode between HCTR2 and NADPH. However, the polar solvation energy is comparatively lower. The higher electrostatic energy of HCTR2-NADPH complex could be correlated to the greater number of charged residues surrounding NADPH. The surface electrostatic potential of the residues present around 4 Å of the cofactor was calculated to find out the reason behind the observed differences in binding free energy components. It was found that in HCTR1 the NADPH was surrounded by almost equivalent number of negatively and positively charged residues, as indicated by the similar sizes of the red and blue surfaces around NADPH (Figure 6). In contrast, the NADPH in HCTR2 was surrounded by more number of negatively charged residues. This is probably the reason why we found increased coulombic terms for HCTR2-NADPH complex. Hence, it could be argued that the distribution of charged residues around the cofactor could affect its affinity with which it binds the enzyme. Earlier studies on NADPH-dependent enzymes revealed that the cofactor binding affinity might affect the specific recognition and catalysis of the HC-toxin [52], [53].

**Table 3:** Binding free energy calculation of enzyme-cofactor complexes (HCTR1-NADPH and HCTR2-NADPH).

Energy Term	HCTR1-NADPH	HCTR2-NADPH
ΔG_bind_	−616.989+/−186.983	−16.9749+/−70.3229
ΔG_coul_	82.733+/−183.07	−787.719+/−111.78
ΔG_ps_	157.814+/−183.026	992.339+/−124.714
ΔG_polar_	240.547	204.62
ΔG_vdW_	−821.868+/−1828.45	−328.679+/−137.446
ΔG_nps_	−30.6304+/−1.13497	−31.801+/−0.432459
ΔG_nonpolar_	−852.498	−360.48

ΔG*_bind_*  =  Binding free energy.

ΔG*_coul_*  =  Electrostatic energy.

ΔG*_ps_*  =  Polar solvation energy.

ΔG*_polar_*  =  Polar term (ΔG*_coul_* + ΔG*_ps_*).

ΔG*_vdW_*  =  van der Waals energy.

ΔG*_nps_*  =  Nonpolar solvation energy.

ΔG*_nonpolar_*  =  Nonpolar term (ΔG*_vdW_* + Δ*_Gnps_*).

#### Identification of HC-Toxin binding residues

**Figure 7:**
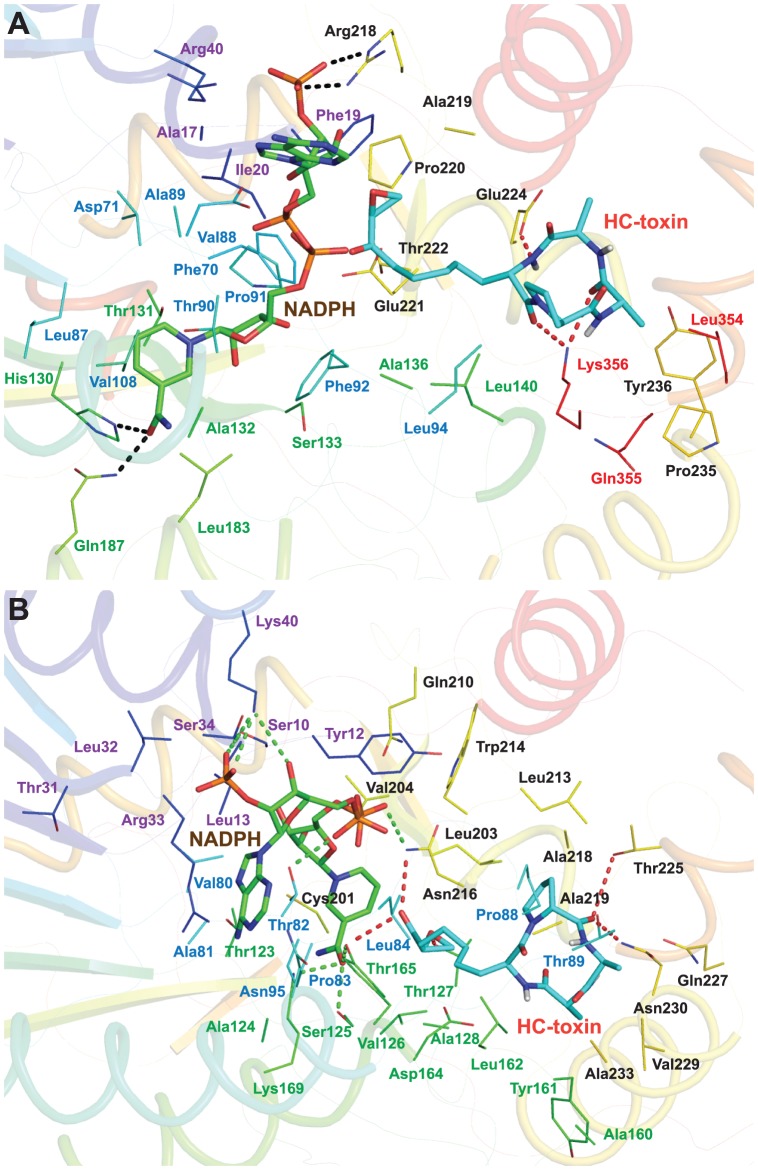
Intermolecular interaction observed between HC-toxin and the HCTR1-NADPH and HCTR2-NADPH complexes. (A) Interaction of HC-toxin with the HCTR1-NADPH complex. The H-bonds formed between HCTR1-NADPH have been marked in black dotted lines whereas H-bonds formed between HCTR1and HC-toxin have been marked in red. (B) Interaction of HC-toxin with the HCTR2-NADPH complex. The H-bonds formed between HCTR2-NADPH has been marked in green dotted lines whereas H-bonds formed between HCTR2 and HC-toxin has been marked in red dotted lines.

Molecular docking of HC-toxin was performed on the final cofactor-docked complexes of HCTRs obtained from MD trajectories. The estimated binding affinity of HC-Toxin towards HCTR1-NADPH complex was found to be −7.70 kcal/mol whereas that towards HCTR2-NADPH was −8.50 kcal/mol (Table 4). A closer observation revealed that the HC-toxin in HCTR1 prefers to bind at a position that is close to the docked NADPH structure (Figure 7A). The OE1 atom of Glu224 forms H-bond with H46 atom of HC-toxin with a interatomic distance of 2.16 Å. The oxygen atoms (O6 and O1) of HC-toxin form two H-bonds with NZ atom of Lys356 with interatomic distances of ∼2.7 Å respectively. Furthermore, HC-toxin was seen entangled in a hydrophobic pocket deep within the HCTR1 enzyme lined by residues Phe71, Phe92, Leu94, Arg218, Glu221, Tyr236 and Gly357 (Figure S5A in File S1). The HC-Toxin prefers to bind HCTR2 in a different orientation compared to HCTR1, where D-pro of HC-toxin lines opposite to the clusters of hydrophobic amino acids (Figure 7B).Thr127 of HCTR2 formed a single H-bond with oxygen (O2) atom of HC-toxin with a distance of 2.6 Å and Asn216 and Asn230 bonded with oxygen (O5 and O3) atoms the HC-toxin. In addition, Leu62, Leu85, Ala128, Asp164, Tyr165, Gly215, Ala219, and Val229 form a tight network of hydrophobic interaction with HC-toxin (Figure S5B in File S1).

**Table 4:** Autodock scores obtained after docking HC-toxin into HCTR1-NADPH (A) and HCTR2-NADPH (B) complexes.

(A)
Rank	Estimated Free Energy of Binding (kcal/mol) [ = (1)+(2)+(3)−(4)]	Estimated Inhibition Constant, Ki	Final Intermolecular Energy (1)	vdW + Hbond + desolv Energy	Electrostatic Energy	Final Total Internal Energy (2)	Torsional Free Energy (3)	Unbound System's Energy (4)
1	−6.11	32.97 uM	−8.20	−7.33	−0.87	−0.56	+2.09	−0.56
2	−5.83	53.67 uM	−7.91	−7.73	−0.19	−0.59	+2.09	−0.59
3	−6.66	13.17 uM	−8.75	−8.09	−0.66	−0.41	+2.09	−0.41
4	−7.01	7.27 uM	−9.10	−8.76	−0.34	−0.56	+2.09	−0.56
5	−7.51	3.11 uM	−9.60	−8.84	−0.76	−0.49	+2.09	−0.49
6	−5.71	64.71 uM	−7.80	−7.52	−0.28	−0.50	+2.09	−0.50
7	−6.27	25.35 uM	−8.36	−7.63	−0.73	−0.46	+2.09	−0.46
8	−7.32	4.32 uM	−9.41	−9.04	−0.36	−0.62	+2.09	−0.62
**9**	−**7.70**	**2.28 uM**	−**9.79**	−**9.32**	−**0.47**	−**0.40**	**+2.09**	−**0.40**
10	−6.31	23.67 uM	−8.40	−7.51	−0.88	−0.51	+2.09	−0.51

(B)
Rank	Estimated Free Energy of Binding (kcal/mol) [ = (1)+(2)+(3)−(4)]	Estimated Inhibition Constant, Ki	Final Intermolecular Energy (1)	vdW + Hbond + desolv Energy	Electrostatic Energy	Final Total Internal Energy (2)	Torsional Free Energy (3)	Unbound System's Energy (4)
1	−8.30	821.56 nM	−10.39	−10.31	−0.08	−0.42	+2.09	−0.42
2	−7.50	3.19 uM	−9.59	−9.68	+0.09	−0.24	+2.09	−0.24
3	−7.08	6.43 uM	−9.17	−8.94	−0.23	−1.01	+2.09	−1.01
4	−7.19	5.36 uM	−9.28	−9.12	−0.15	−0.64	+2.09	−0.64
5	−7.66	2.44 uM	−9.75	−9.68	−0.07	−0.60	+2.09	−0.60
6	−7.57	2.83 uM	−9.66	−9.54	−0.12	−0.69	+2.09	−0.69
**7**	−**8.50**	**592.28 uM**	−**10.58**	−**10.52**	−**0.07**	−**0.58**	**+2.09**	−**0.58**
8	−7.11	6.18 uM	−9.19	−9.16	−0.03	−0.12	+2.09	−0.12
9	−8.00	1.36 uM	−10.09	−10.06	−0.03	−0.59	+2.09	−0.59
10	−7.35	4.10 uM	−9.44	−9.21	−0.23	−0.35	+2.09	−0.35

## Conclusions

In this study, we have modeled and predicted the interaction between the cofactor, NADPH and two disease resistance enzymes, HCTR1 and HCTR2 of maize plant using molecular docking and MD simulations. MM/PBSA binding free energy calculations revealed that the cofactor binding sites within the enzymes are distinct. HCTR1 mainly recruits nonpolar residues whereas HCTR2 prefers polar residues to bind the NADPH. The binding modes of NADPH on the two HCTRs were found to be energetically different. The overall stability of HCTR1's active site depends on van der Waals interaction with the cofactor, while the HCTR2's active site was stabilized by electrostatic interactions with the cofactor. Our study also highlighted the role of number of H-bonds electrostatic contacts for maintaining the HCTR-NADPH interactions. In addition, we predicted the possible HC-toxin binding residues in enzymatic class of resistance genes, which can be considered suitable for future site-directed mutagenesis studies. We expect our findings have the potential to be translated further through biochemical and structural biology studies that will significantly aid in achieving durable resistance in plants, thereby contributing to the global food security.

## Supporting Information

File S1 **Contains Text S1 and S2, Figures S1–S5 and Tables S1–S5.** Text S1. Description of model validation scores. Text S2. Description of ligand conformation generation and scoring functions considered for molecular docking. Figure S1. Ramachandran and ProSA plots of modeled HCTR1 and 2. Figure S2. Superimposition of built models over the templates. Figure S3. Interaction between HCTRs and NADPH. Figure S4. Secondary structure deviation as a function of simulation time. Figure S5. 2D representation of interaction between HC-toxin and HCTRs-NADPH complexes. Table S1. The atomic composition of the HCTRs-NADPH simulation systems. Table S2. Energy components derived from “calculate binding energy” protocol in DS3.5 where the best 10 ligand poses (NADPH) for HCTR1 are scored. Table S3. Final energy terms for best pose of NADPH for HCTR1 and HCTR2 respectively derived from DS3.5. Table S4. The consensus scoring scheme used for various poses of the best 10 poses of cofactor NADPH with modeled (A) HCTR1 and (B) HCTR2. Ligand poses are scored using ‘score ligand poses’ protocol in DS3.5. Table S5. H-bond interacting residues with their atomic components obtained after MD simulation of HCTR1–NADPH complex (A) and HCTR2–NADPH complex (B).
(PDF)
