# Supplementary material for: Structure-Based Computational Study of Two Disease Resistance Gene Homologues (Hm1 and Hm2) in Maize (Zea mays L.) with Implications in Plant-Pathogen Interactions
Source: PLoS One. 2014 May 21;9(5):e97852. doi: 10.1371/journal.pone.0097852 (PMC4029905; doi:10.1371/journal.pone.0097852)
Supplement: File S1 — Contains Text S1 and S2, Figures S1–S5 and Tables S1–S5. Text S1. Description of model validation scores. Text S2. Description of ligand conformation generation and scoring functions considered for molecular docking. Figure S1. Ramachandran and ProSA plots of modeled HCTR1 and 2. Figure S2. Superimposition of built models over the templates. Figure S3. Interaction between HCTRs and NADPH. Figure S4. Secondary structure deviation as a function of simulation time. Figure S5. 2D representation of interaction between HC-toxin and HCTRs-NADPH complexes. Table S1. The atomic composition of the HCTRs-NADPH simulation systems. Table S2. Energy components derived from “calculate binding energy” protocol in DS3.5 where the best 10 ligand poses (NADPH) for HCTR1 are scored. Table S3. Final energy terms for best pose of NADPH for HCTR1 and HCTR2 respectively derived from DS3.5. Table S4. The consensus scoring scheme used for various poses of the best 10 poses of cofactor NADPH with modeled (A) HCTR1 and (B) HCTR2. Ligand poses are scored using ‘score ligand poses’ protocol in DS3.5. Table S5. H-bond interacting residues with their atomic components obtained after MD simulation of HCTR1–NADPH complex (A) and HCTR2–NADPH complex (B). (PDF) [file pone.0097852.s001.pdf]

Supporting Information

Structure-based computational study of two disease resistance gene homologues (*Hm1* and *Hm2*) in maize (*Zea mays* L.) with implications in plant-pathogen interactions

Budheswar Dehury[1,2±], Mahesh Chandra Patra[3,4±], Jitendra Maharana[3,5], Jagajjit Sahu[1], Priyabrata Sen[1], Mahendra Kumar Modi[1], Manabendra Dutta Choudhury[2] and Madhumita Barooah[1*]

[1]Department of Agricultural Biotechnology, Assam Agricultural University, Jorhat-785013, Assam, India

[2]Department of Life Science and Bioinformatics, Assam University, Silchar-788011, Assam, India

[3]BIF-Centre, Department of Bioinformatics, Orissa University of Agriculture and Technology, Bhubaneswar-751003, Odisha, India

[4]Animal Genomics Laboratory, Animal Biotechnology Centre, National Dairy Research Institute, Karnal-132001, Haryana, India

[5]Biotechnology Laboratory, Central Inland Fisheries Research Institute, Barrackpore, Kolkata-700120, West Bengal, India

***Corresponding author**

Madhumita Barooah

Department of Agricultural Biotechnology

Assam Agricultural University, Jorhat-785013, Assam

Email: m17barooah@yahoo.co.in

Tel: +91-(0376)-2340095 (O)

Fax: +91 0376 2340001/2340101

[±]Contributed equally to the work

Text S1: Description of model validation scores.

Procheck analysis revealed that 90.4 and 90.2% of residues of HCTR1 and HCTR2, respectively, fall under the most favored region of the Ramachandran plot (Figure S1). The overall G-factors of HCTR1 and HCTR2 were 0.19 and -0.14 (well below the cut-off score of 0.5) (Table 1). No residues were found within the disallowed region of the plot, indicating good stereo-chemical quality. The ERRAT scores of HCTR1 and HCTR2 were 71.38 and 78.9%, respectively, which are far greater than the cut-off 50% [1]. ERRAT provides accuracy of the non-bonded atomic contacts. The Verify 3D scores of HCTR1 and HCTR2 were 95.81 and 94.97%, respectively, which are greater than the cut-off value of 80% [2], indicating the built models are of good qualities. The ProSA z-scores of the models were highly correlated with their templates, indicating good quality [3] (Table 1 and Figure S1). Further evaluation of the models is summarized in Table 1, suggesting the validation scores are highly comparable with or better than templates.

Text S2: Description of ligand conformation generation and scoring functions considered for molecular docking.

Initially both of the proteins and ligand (NADPH) were prepared in DS3.5. About 200 different conformations of NADPH were generated using high temperature molecular dynamics simulations. Out of 200, only five conformations of NADPH with the lowest energies were considered the potential candidates for docking. The high temperature MD simulation offers all the advantages of full ligand flexibility (including bonds, angles and dihedrals) and reasonable computation time. Various empirical scoring functions such as LigScore (LigScore1 and LigScore2) [4], PLP (PLP1, and PLP2) [5] and knowledge-based scoring functions like Jain (Jain 1996) [6], PMF (PMF and PMF04) [7, 8] along with energy functions CDOCKER ENERGY, CDOCKER_Interaction Energy [9] was used to rank NADPH poses within HCTRs (Supporting information Table S4). The top candidate binders from the best selected 10 poses, as judged by consensus scoring, were used for further enzyme-cofactor molecular dynamics studies.

Supporting Figures

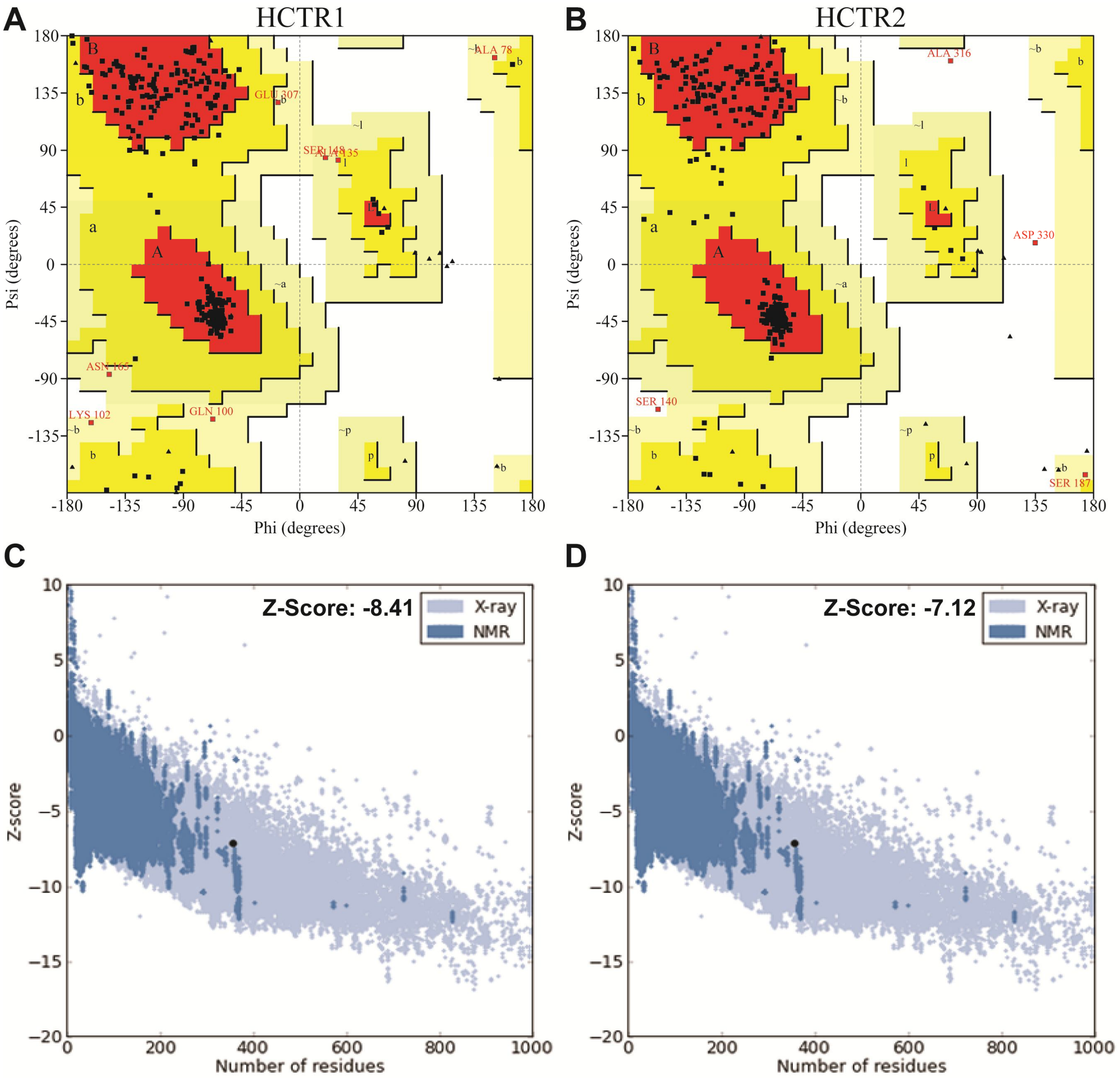

Figure S1:Ramachandran and ProSA plots of modeled HCTR1 and 2.

Ramachandran plots of (A) HCTR1, (B) HCTR2. ProSA plots of (C) HCTR1, (D) HCTR2.

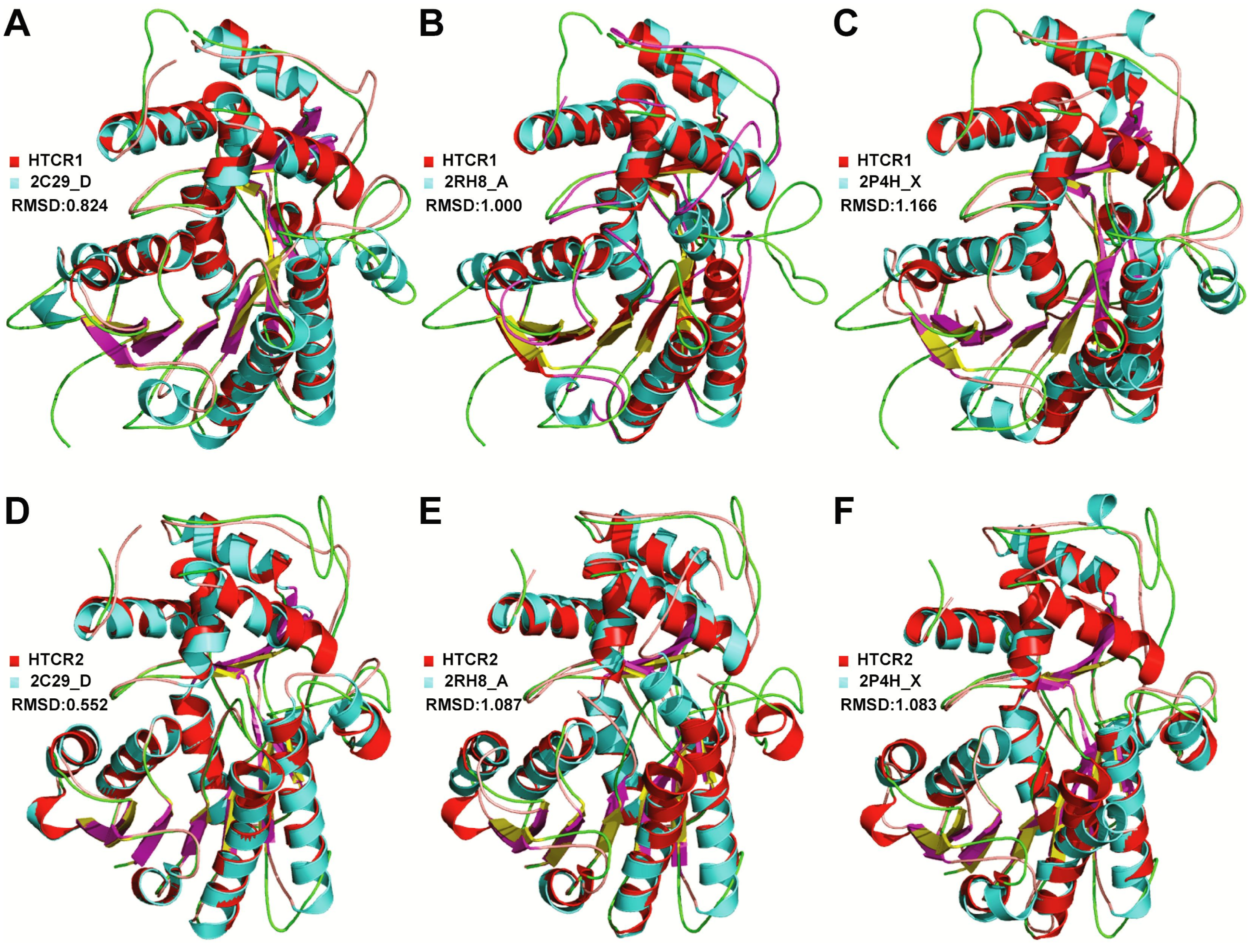

Figure S2: **Superimposition of built models over the templates.**

Pair-wise structural superposition of Cα atoms of the modeled HCTRs with their respective templates (PDB ID: 2C29, 2RH8 and 2P4H)

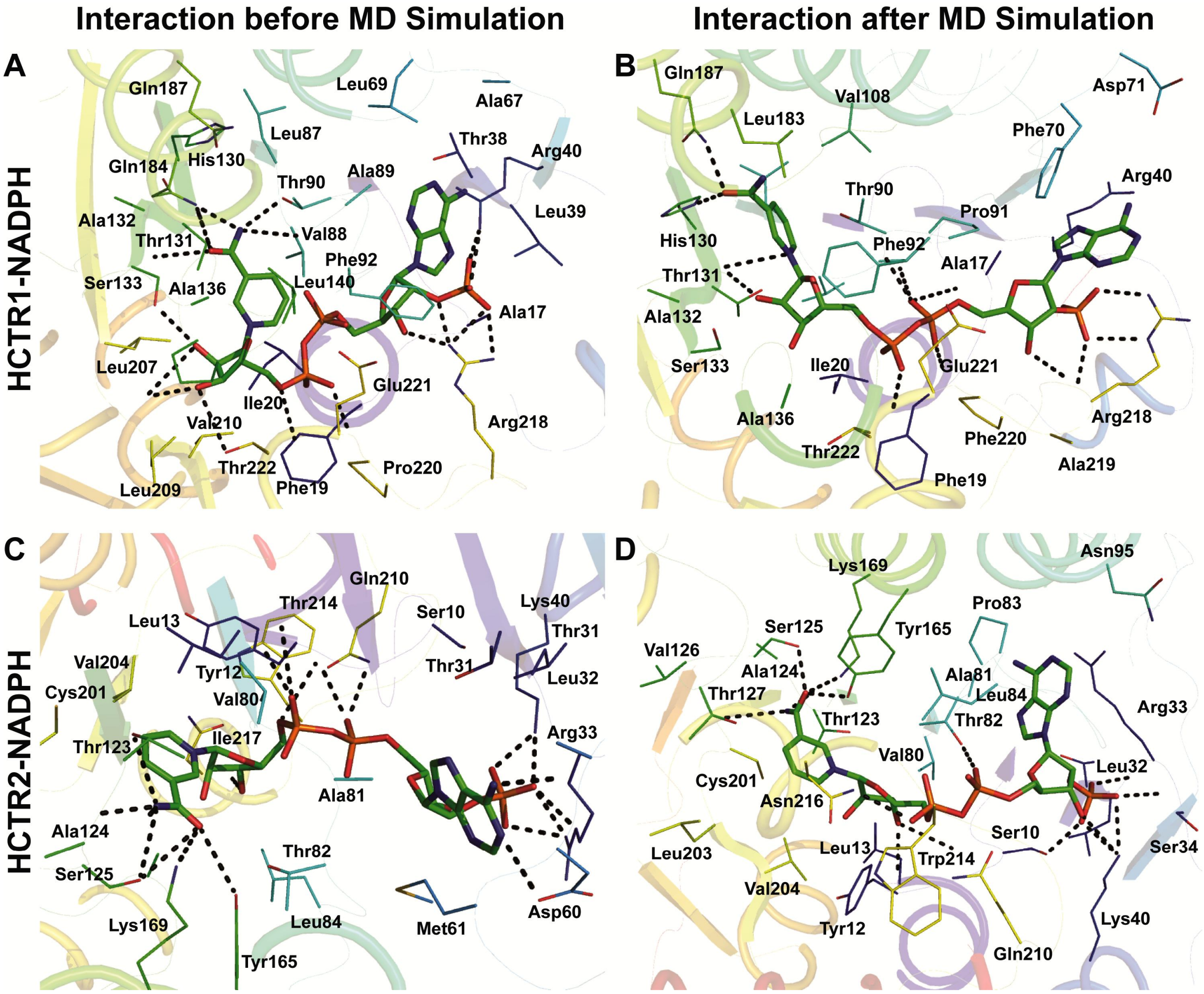

Figure S3: Interaction between HCTRs and NADPH.

The representation of intermolecular contacts between the modeled HCTRs and the NADPH before (A and C) and after (B and D) MD simulations. Hydrogen bonds are marked with black dotted lines where as other interacting residues are labeled in lines.

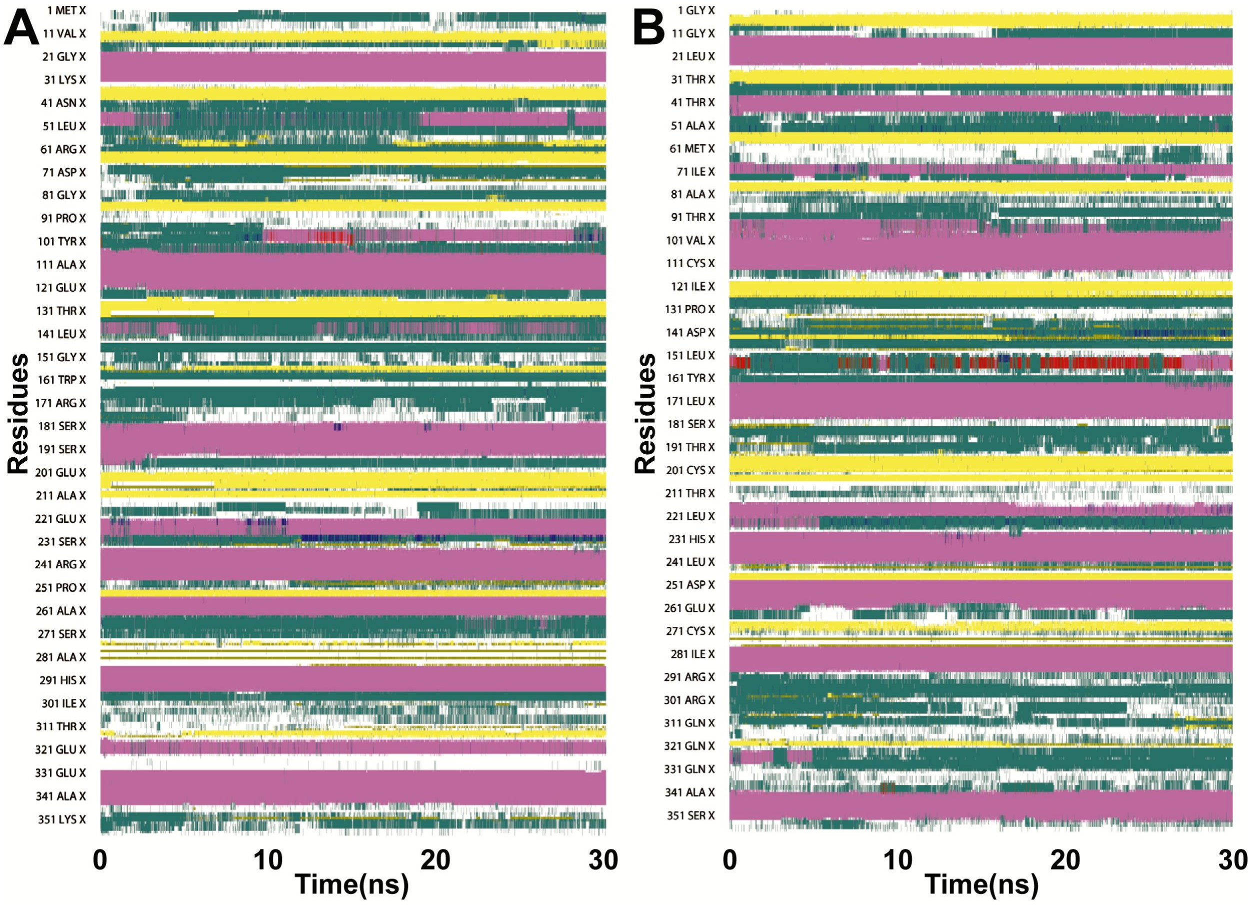

Figure S4: Secondary structure deviation as a function of simulation time.

Secondary structure deviations as a function of simulation time in the modeled HCTRs-NADPH complexes, indicating the conservation of secondary structures throughout the 30-ns MD simulation. (A) HCTR1-NADPH (B) HCTR2-NADPH complexes.

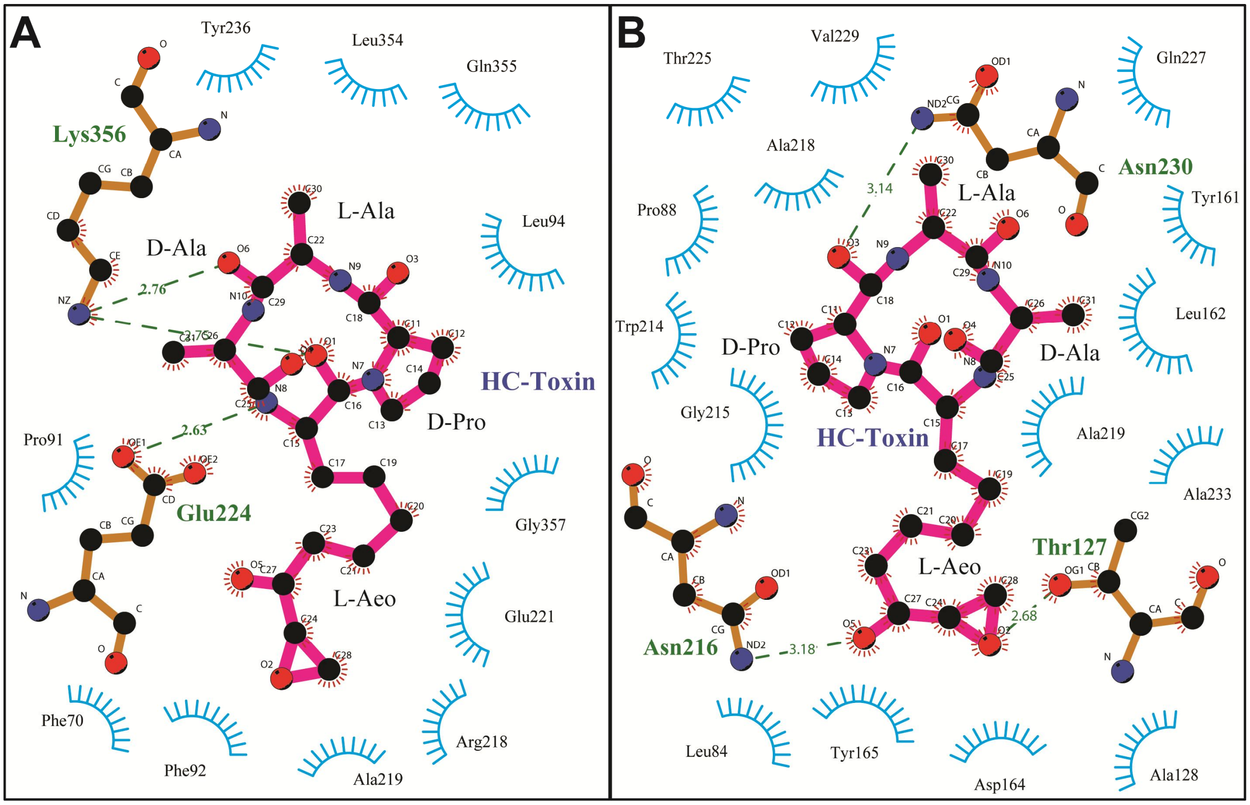

Figure S5: 2D representation of interaction between HC-toxin and HCTRs-NADPH complexes. The intermolecular interactions of HC-toxin with (A) HCTR1-NADPH and (B) HCTR2-NADPH docked complexes. The image was generated using LigPlot+ software; HC-Toxin is shown in magenta; H-bond forming amino acids are labeled in green, and the hydrophobic contacts are labeled in light blue half circles.

Supplementary Tables

Table S1

The atomic composition of the HCTRs-NADPH simulation systems.

Simulation system (Enzyme-cofactor complex)	Number of amino acids (atoms)	Number of atoms in cofactor (NADPH)	Number of solvent molecule	Number of Na^+ atoms	Number of Cl^- atoms	Total number of atoms
HCTR1-NADPH	357 (3475)	61	13362	52	42	43693
HCTR2-NADPH	357 (3475)	61	12526	45	39	41198

Table S2

Energy components derived from "calculate binding energy" protocol in DS3.5 where the best 10 ligand poses (NADPH) for HCTR1 are scored.

A

Pose No	Binding energy (kcal/mol)	Ligand energy (kcal/mol)	Protein energy (kcal/mol)	Complex energy (kcal/mol)	Entropic Energy (kcal/mol)
1	-428.679	-235.239	-9,458.61	-10,122.50	-33.0932
2	-229.855	-173.193	-9,458.61	-9,861.66	-33.0932
3	-164.27	-138.626	-9,458.61	-9,761.51	-33.0932
4	675.59	-209.406	-9,458.61	-8,992.43	-33.0932
5	-668.904	-120.381	-9,458.61	-10,247.90	-33.0932
6	-553.576	-93.2512	-9,458.61	-10,105.40	-33.0932
7	-572.503	-109.485	-9,458.61	-10,140.60	-33.0932
8	-266.166	-119.096	-9,458.61	-9,843.87	-33.0932
9	-114.006	-119.901	-9,458.61	-9,692.52	-33.0932
10	-128.089	-123.577	-9,458.61	-9,710.28	-33.0932

B

Pose No	Binding Energy (kcal/mol)	Ligand Energy (kcal/mol)	Protein Energy (kcal/mol)	Complex Energy (kcal/mol)	Entropic Energy (kcal/mol)
1	-505.38869	-157.4016	-10011	-10674.19166	23.6358
2	-518.28678	-124.10057	-10011	-10653.78872	23.3864
3	-418.30202	-146.14535	-10011	-10575.84874	23.7266
4	-310.05473	-166.23498	-10011	-10487.69108	23.4454
5	-593.6229	-67.10228	-10011	-10672.12655	23.5631
6	-621.9196	-127.50801	-10011	-10760.82898	23.6736
7	-581.01973	-74.63112	-10011	-10667.05222	23.5912
8	-661.63793	-89.62461	-10011	-10762.66391	23.5625
9	-591.63276	-109.94663	-10011	-10712.98076	23.6346
10	-662.93253	-93.27046	-10011	-10767.60436	23.7178

Table S3

Final energy terms for best pose of NADPH for HCTR1 and HCTR2 respectively derived from DS3.5.

Protein Name	Cofactor	Binding Energy (kcal/mol)	Ligand Energy (kcal/mol)	Protein Energy (kcal/mol)	Complex Energy (kcal/mol)	Entropic Energy (kcal/mol)
HCTR1	NADPH	-428.6306	-235.24485	-9458.5	-10122.359	23.5822
HCTR2	NADPH	-572.39531	-112.57077	-10011.0	-10696.358	23.7258

Table S4

The consensus scoring scheme used for various poses of the best 10 poses of cofactor NADPH with modelled (A) HCTR1 and (B) HCTR2. Ligand poses are scored using score ligand poses protocol in DS3.5.

A

Pose No	CODCKER energy	CODCKER Intercation Energy	LigScore1 _Dreiding	LigScore2 _Dreiding	PLP1	PLP2	Jain	PMF	PMF04
1	46.445	67.993	5.82	6.95	122.48	106.78	6.16	95.71	28.57
2	46.212	66.969	5.48	6.84	119.34	103.64	6.47	90.5	23.57
3	46.044	67.258	5.6	6.9	118.86	103.22	6.07	90.1	23.51
4	44.143	68.696	5.72	6.42	123.28	107.76	7.86	89.47	20.48
5	39.126	60.919	4.83	6.17	110.53	99.08	6.18	77.37	6.74
6	36.804	62.028	4	5.76	111.81	97.6	5.53	89.39	22.31
7	32.701	55.753	4.07	5.41	114.74	102.48	8.68	68.44	3.6
8	32.143	65.24	5.36	6.54	117.36	106.62	6.64	86.92	19.76
9	30.837	55.376	3.98	5.4	110.79	102.4	6.94	72.75	8.08
10	29.156	58.28	4.27	5.97	107.34	91.98	5.83	79.92	11.65

B

Pose No	CODCKER energy	CODCKER Intercation Energy	LigScore1 _Dreiding	LigScore2 _Dreiding	PLP1	PLP2	Jain	PMF	PMF04
1	87.93	109.085	6.88	7.52	120.39	112.48	3.18	76.44	18.09
2	77.354	104.443	7.59	7.67	126.49	123.24	5.93	81.77	23.81
3	62.81	92.445	7.69	7.94	115.11	105.24	1.45	97.38	35
4	60.993	85.339	8.12	7.49	119.6	112.59	4.42	42.33	7.52
5	88.339	114.492	8.15	7.95	120.11	107.67	3.54	112.5	42.82
6	88.27	115.213	8.21	8.24	135.9	128.52	5.43	116.18	48.91
7	87.548	115.159	8.03	7.76	120.28	102.63	2.14	109.22	37.72
8	87.318	115.963	8.58	8.56	141.88	124.71	5.73	105.77	35.42
9	86.352	112.267	8.09	8.24	122.98	114.81	2.3	97.82	39.56
10	80.709	111.904	7.84	8.1	113.31	97.49	2.21	100.87	52.65

Table S5

H-bond interacting residues with their atomic components obtained after MD simulation of HCTR1–NADPH complex (**A**) and HCTR2–NADPH complex (**B**).

A

Sl. No	Residue	Donor atom (HCTR1)	Acceptor atom (NADPH)	Atomic distance
1	Arg218	HH21 (SC)	O47 (PO4)	2.1
2	Arg218	HE (SC)	O46 (PO4)	2.3
3	Arg218	H	O46 (PO4)	1.8
4	Arg218	O	H9	2.3
5	Thr90	H	O3 (PO4)	2.1
6	Thr222	H	O25 (PO4)	1.9
7	Phe19	H	O2	2.0
8	Thr131	O	H34	2.5
9	His130	HE2 (SC)	O40	1.6
10	Gln187	HE22 (SC)	O40	1.7
11	Arg40	HE	N15	2.3

B

Sl. No	Residue	Donor atom (HCTR2)	Acceptor atom (NADPH)	Atomic distance
1	Ser125	HG (SC)	O40	1.5
2	Lys169	HZ1 (SC)	O40	2.2
3	Asn216	HD22 (SC)	O26	2.3
4	Thr82	HG1 (SC)	O2	2.0
5	Leu13	H	O32	2.3
6	Ser10	HG (SC)	O47	1.6
7	Lys40	HZ1 (SC)	O48	2.3
8	Ser34	H	O48	2.2
9	Arg33	H	O46	1.8
10	Gln210	HE22	O26	2.7
11	Ala124	O	H7	2.3
12	Thr82	HN	N15	2.0
13	Tyr165	HH	O40	2.5

*SC stands for side chain of corresponding interacting amino acids

References

1. Colovos C, Yeates TO (1983) Verification of protein structures: patterns of nonbonded atomic interactions. Protein Science 2: 1511–1519.
2. Eisenberg D, Luthy R, Bowie JU (1997) VERIFY3D: assessment of protein models with three-dimensional profiles. Methods in Enzymology 277: 396–404.
3. Wiederstein M, Sippl MJ (2007) ProSA-web: interactive web service for the recognition of errors in three-dimensional structures of proteins. Nucleic Acids Research 35: W407–W410.
4. Krammer A, Kirchhoff PD, Jiang X, Venkatachalam CM, Waldman M (2005) LigScore: a novel scoring function for predicting binding affinities. Journal of Molecular Graphics and Modeling 23: 395–407.
5. Gehlhaar DK, Verkhivker GM, Rejto PA, Sherman CJ, Fogel DB, Fogel LJ, Freer ST (1995) Chemical Biology 2: 317–324.
6. Jain AN (1996) Scoring noncovalent protein-ligand interactions: a continuous differentiable function tuned to compute binding affinities. Journal of Computer Aided Molecular Design 10: 427-440.
7. Muegge I (2006) PMF scoring revisited. Journal of Medicinal Chemistry 49: 5895-5902.
8. Muegge I, Martin YC (1999) A general and fast scoring function for protein-ligand interactions: a simplified potential approach. Journal of Medicinal Chemistry 42: 791–804.
9. Huang N, Kalyanaraman C, Bernacki K, Jacobson MP (2006) Molecular mechanics methods for predicting protein-ligand binding. Physical Chemistry Chemical Physics 8: 5166–5177.
